# Testing the efficacy of tocilizumab in patients with COVID-19 pneumonia

**DOI:** 10.2217/cer-2021-0050

**Published:** 2021-04-23

**Authors:** Mehmet Agirbasli, Azra Tanrıkulu

**Affiliations:** ^1^Department of Cardiology, Medeniyet University Medical School, Istanbul, Turkey; ^2^Department of Cardiology, Maltepe State Hospital, Istanbul, Turkey

**Keywords:** clinical trials, COVID-19, tocilizumab

Coronavirus infection (COVID-19) is affecting societies and economies globally. Developing therapies for hospitalized patients is therefore a matter of global urgency, in order to optimize care and outcomes. Investigators are working to identify molecular targets with a potential role in the development and outcome of adult respiratory distress syndrome in COVID-19, seeking pragmatic, novel and rational targets. This is being followed by rapidly designed randomized controlled trials (RCTs), with the subsequent aim of developing population-based recomendations. The common end points for this research include preventing intubation or death in hospitalized patients with COVID-19.

In this context, the efficacy of tocilizumab in patients with COVID-19 has been examined in several randomized trials [1–6]. The trials differ significantly in their inclusion and exclusion criteria, enrolling patients with different disease severity at baseline. Commonly, researchers have reported that the confidence interval (CI) for efficacy comparisons are wide, so benefit or harm of treatment is uncertain.

The trials did not show a difference in mortality between the tocilizumab and control groups. The efficacy end point of survival without invasive or noninvasive mechanical ventilation by day 14 was achieved in the CORIMUNO-TOCI-1 trial [4]; however, without a mortality benefit at day 28. The results of the trials suggest that patient enrollment criteria determine the benefit experienced with tocilizumab. Due to wide CIs, the conclusion is made in these trials that tocilizumab does not show efficacy.

CIs are helpful in determining not only statistical significance but also the clinical relevance of findings. Hospitalized patients with COVID-19 display wide physiological and biological heterogeneity [7]. Wide CIs suggest how large the effect of a treatment could plausibly be. Thus, we cannot rule out the possibility that tocilizimab would help in certain groups of patients. In fact, a systematic review and meta-analysis reports that addition of tocilizumab to standard of care reduces mortality in severe COVID-19 [8].

On the other hand, a recent trial was stopped early by the data monitoring committee because of an increased number of deaths at 15 days in the tocilizumab group. This trial enrolled 129 patients with severe or critical COVID-19 [9]. The study population included adults with confirmed COVID-19 who were receiving supplemental oxygen or mechanical ventilation and had abnormal levels of at least two serum biomarkers (CRP, D dimer, LDH or ferritin).

However, the results from the Randomized, Embedded, Multifactorial Adaptive Platform Trial for Community-Acquired Pneumonia (REMAP-CAP) found that in critically ill patients with COVID-19 requiring support in the ICU, treatment with the tocilizumab improved survival [10].

On 3 February 2021, the COVID-19 Treatment Guidelines Panel (the Panel) of NIH issued a statement on the use of tocilizumab in certain group of patients with COVID-19 based on the collective evidence from the REMAP-CAP and Randomized Evaluation of COVID-19 Therapy (RECOVERY) trials [11]. The results of the RECOVERY and REMAP-CAP trials found that tocilizumab, when added to corticosteroid therapy, offers a modest mortality benefit in patients with COVID-19 who exhibit rapid clinical deterioration with increasing oxygen needs and a significant inflammatory response to the virus. The Panel recommended the use of tocilizumab in combination with dexamethasone in these patients. The criteria for a potential benefit from tocilizumab are: recently hospitalized patients who have been admitted to the ICU within the prior 24 h and who require invasive mechanical ventilation, noninvasive mechanical ventilation or high-flow nasal canula oxygen; or recently hospitalized patients (not in the ICU) with rapidly increasing oxygen needs who require noninvasive mechanical ventilation or high-flow nasal canula and have significantly increased markers of inflammation (inclusion criteria was CRP ≥75 mg/l).

In conclusion, we believe that there are lessons to be learned from RCTs testing the efficacy and safety of tocilizumab in COVID-19, in the midst of the global pandemic. Lack of effective surrogate biomarkers of clinical outcome in COVID-19, can at least partially explain the conflicting results in the RCTs. For conditions such as COVID-19, it is important to understand the mechanisms underpinning the heterogeneity in drug-related outcomes. Without further understanding of the basis of individual differences in drug safety and efficacy, it is difficult to draw definitive conclusions, and we run the risk of dismissing potentially useful treatments from further development. As with any maturing field, conflicting observations should be expected. Global efforts are needed to inform health care professionals about the interpretation of conflicting results and messages.

We wish to emphasize, however, that avoiding premature clinical decisions in today’s difficult conditions should be a centerpiece concept in clinical pharmacology and population health. With growing technological advances, effective drugs are being developed and used in COVID-19. Timely detection of population signals pertaining to efficacy and toxicity is therefore very important.

COVID-19 is a current and urgent matter globally. Medical communities could not have anticipated the severity of the situation we are facing today. In the midst of the pandemic we run the risk of visualizing the real effects of therapies too late, leading to premature decisions about safety. Exercising pharmacovigilance and the novel concept of pharmacogenovigilance thorough global centers can offer a mechanistic insight and firm causality assessment of COVID-19 therapies [12,13].

In conclusion, COVID-19 trials are in need of anticipatory systems that can detect early signals of safety and efficacy in the transition of health interventions to population-scale applications. The premature decision to remove a potentially useful therapy from further development could be a risk of today’s difficult conditions.
